# Suprasternal aortic arch echocardioscopy as a potential tool in detection and follow-up of mobile thrombi in patients with ischemic stroke

**DOI:** 10.3389/fneur.2024.1528325

**Published:** 2025-01-07

**Authors:** Inhar Esnaola Barriola, Elena Escriche Gorospe, Paula Miguel Navas, Elisa Martínez Campos, María Molina Goicoechea, Ferran Capell Pascual, Adrián Jiménez Delgado, Roberto Muñoz Arrondo

**Affiliations:** Neurology Department, Navarre University Hospital, Pamplona, Navarra, Spain

**Keywords:** aortic arch, mobile thrombus, suprasternal, echocardioscopy, ischemic stroke, anticoagulation

## Abstract

**Introduction:**

Severe or complicated atheromatosis of the aortic arch represents an important and often underdiagnosed embolic source in patients with ischemic stroke. The presence of a floating thrombus has significant clinical relevance, as it is associated with a high risk of early recurrence. The aim of this study was to analyze the potential of echocardiographic examination through the suprasternal window in both the detection of embolic sources and the monitoring of the response to anticoagulant treatment in patients with mobile thrombi.

**Methods:**

This case series study included ten consecutive patients with a mobile floating aortic arch thrombus associated with an atheromatous plaque, detected by focused echocardiography and confirmed by Computed Tomography Angiography (CTA). Epidemiological, clinical, radiological, and ultrasound characteristics were analyzed. Clinical and ultrasound follow-up was performed after initiation of anticoagulation as secondary prevention to assess the efficacy and safety of this treatment.

**Results:**

Ten patients (seven female) with a mean age of 76 years were identified. After anticoagulation, a complete resolution of the mobile thrombus was observed in eight of them during ultrasound follow-up. One patient suffered an ischemic recurrence. Two patients receiving associated antiplatelet therapy presented severe hemorrhagic complications, one of which was fatal. Once the disappearance of the mobile thrombus was detected, anticoagulation was discontinued, and no further ischemic recurrences were observed.

**Discussion:**

Floating thrombus of the aortic arch is an underdiagnosed but clinically relevant condition. The study of the aortic arch with echocardiography through the suprasternal window is a highly available and harmless technique, that may be highly useful for the detection and monitoring of response to treatment of this pathology. Furthermore, early anticoagulation could be an effective and safe treatment in these patients.

## Introduction

Ischemic stroke can arise from various distinct mechanisms and understanding the underlying cause is crucial, as this knowledge guides evidence based treatment strategies to prevent future strokes (1).

Approximately 25% of ischemic strokes are classified as cryptogenic (2), and about one sixth fit the criteria for Embolic Stroke of Undetermined Source (ESUS), defined as a stroke that appears non-lacunar on neuroimaging without an identifiable source after a comprehensive evaluation to exclude known stroke etiologies (3). The challenge lies in identifying effective secondary prevention strategies when the specific mechanism of the stroke is unknown.

Different studies have highlighted a correlation between Aortic Arch Atherosclerosis (AAA) and cerebral infarction, particularly in those cases where no obvious etiology is identified (4, 5). Nevertheless, the aortic arch presents a challenging area for the diagnostic techniques typically employed in clinical practice. Consequently, there is a growing recognition of this vascular territory as a ‘missing link’ in many cerebral infarctions of undetermined origin, especially evident in patients experiencing recurrent ischemic events despite receiving appropriate general treatment (5).

While transesophageal echocardiography (TEE) has classically been considered the gold standard for detecting AAA and their mobile components, transthoracic echocardiography (TTE) offers valuable visualization of thoracic aorta, including the aortic arch through the suprasternal window, a place where aortic atheroma is common. It is precisely in this area that there is often a blind zone for TEE due to the overlapping of the trachea and the right main bronchus, which does not allow the transesophageal probe to provide images of sufficient resolution (6). Therefore, suprasternal window TTE (sTTE) can effectively visualize protruding atheromas and even mobile components, serving as an excellent screening tool and providing complementary views to TEE examination. Although it is not always possible to obtain an image with sufficient resolution, the available studies obtain images in up to 84% of patients (6, 7), which makes it ideal as a screening technique.

The wide use of CT angiography in potential candidates for thrombectomy (8) has placed this technique in a preferential position in the study of AAA. Its main strengths are short acquisition times and high reproducibility. It also provides information on plaque composition and even on the presence of intraluminal thrombi. However, this modality has limitations, including reduced availability, exposure to ionizing radiation, and the necessity for contrast administration, so it is not an ideal technique for serial studies.

Given the paucity of literature on this subject and the hypothesis that it may be a valuable tool for identifying embolic sources in the aortic arch causing stroke, the objective of this study is to evaluate the potential of sTTE as a promising technique in the diagnosis and monitoring of mobile aortic thrombi in patients with ischemic stroke.

## Materials and methods

We describe a consecutive series of ten patients with mobile floating aortic arch thrombi associated with atheromatous plaques, visualized by sTTE. Data collection was conducted between 2017 and 2024 at a Spanish tertiary referral center (Navarre University Hospital).

The sTTE studies were performed by an experienced vascular neurologist (RMA) certified in focused stroke echocardiography according to the Spanish Certification Consensus (9). Patients who underwent an echocardiographic study, as part of the etiological evaluation of the stroke, that detected a mobile thrombus in the suprasternal window, and whose findings were subsequently confirmed by CT angiography, were included in the study. Patients who did not meet all these criteria were excluded.

Echocardiographic data were acquired using Philips Affinity CV and Philips CX50 equipment (Philips Ultrasound Systems, Bothell, WA) with a 2–5 MHz sector array ultrasound transducer probe. Patients were positioned supine, and the window was deemed satisfactory when the aortic arch image was captured in its long axis, encompassing the origins of the innominate, left common carotid, and left subclavian arteries. The study was complemented with parasternal, apical, and subcostal views. Additionally, each study was supplemented with CTA, providing information about thrombus number, localization, morphology, and mobility.

Data collected from hospital records included patient demographics, personal and family medical history, cardiovascular and thrombotic risk factors, iatrogenic causes, proinflammatory states, other embolic events, atherosclerotic burden, functional status, clinical presentation, diagnostic method, stroke pattern, and treatment. Patient follow-up comprised regular outpatient clinic visits and ultrasound monitoring to assess evolution and response to treatment. Individual patient consent was obtained, and the study was conducted in accordance with the requirements of the local ethics committee.

## Results

### Patient characteristics

Over a period of eight years, a total of ten patients with aortic arch thrombus were identified. Patient characteristics and risk factors are presented in Table 1.

**Table 1:** Patient characteristics and risk factors.

*n*	Age	Sex	Cardiovascular risk factors	Procoagulant abnormalities or Proinflamatory states	Iatrogenic causes	Atherosclerotic load
1	83	F	HT, DL	Normal	-	Severe abdominal Mild carotid
2	79	F	HT, DL	Normal	Cardiac catheterization	Moderate coronary
3	81	F	-	anti-B2GP1 antibodies	-	Mild carotid
4	84	F	-	Not tested	-	Mild carotid
5	75	F	Smoking, DL	Not tested	Cardiac catheterization	Moderate coronary Severe carotid Severe aortic
6	87	F	DM, DL	Normal	-	Mild carotid Severe intracraneal
7	70	M	HT, DL, Smoking	Normal	-	Severe carotid Mild intracraneal
8	79	F	DL	Not tested Giant cell arteritis	Corticosteroid therapy	Mild carotid Mild intracraneal
9	67	M	DM, Smoking	Normal	-	Moderate peripheric Severe aortic Severe coronary Severe carotid
10	53	M	HT, DL, Smoking	Factor V Leiden	-	Mild carotid Mild intracraneal

F, Female; M, Male; HT, Hypertension; DL, Dyslipedemia; DM, Diabetes Mellitus.

Seven patients were female, with a mean age of 76 years (range, 53–87 years). All patients presented with an ischemic vascular event and underwent TTE through the in the acute phase, which detected the presence of a mobile thrombus associated with an atheromatous plaque, in the absence of another embolic source.

In all patients, a review was made of factors that could favor the presence of aortic atheromatosis, including: the presence of vascular risk factors, a high atherosclerotic burden at other levels, underlying procoagulant states, or relevant family history.

Regarding vascular risk factors, the majority of patients (*n* = 8) presented with at least one vascular risk factor: hypertension (*n* = 4), dyslipidemia (*n* = 7), smoking (*n* = 4), diabetes mellitus (*n* = 2) and COPD (*n* = 1). Seven patients had at least two vascular risk factors, and three had three or more.

In relation to the presence of systematic atheromatosis, all patients had atherosclerotic plaques at other levels. Two patients had atheromatous lesions with stability criteria and no signs of severe stenosis and the rest had atheromatosis with severity data. Eight of the patients had atheromatosis in other localization. Three had coronary artery disease, two of them with a previous history of acute myocardial infarction with multivessel disease. One patient had an occlusion of the superior mesenteric artery by atherosclerotic plaque. Six had carotid stenosis >70% or stenosis in intracranial arteries. There was no evidence of systemic embolism in any of the patients.

For the study of procoagulant conditions, thrombophilia testing was performed in 6 patients, including tests for lupus anticoagulant, protein C activation resistance, functional protein C and protein S, functional antithrombin, factor VIII, anti-cardiolipin antibodies (IgG and IgM), anti-β2 glycoprotein 1 antibodies (IgG and IgM), and molecular genetic studies for Factor V Leiden and Prothrombin. One patient was found to have a heterozygous pathogenic polymorphism of Factor V Leiden.

On addition, yartogenic causes that could have mediated an embolic mechanism of stroke due to the detachment of plaques or mobile elements were also reviewed. In two patients, the stroke occurred following cardiac catheterization: one via radial approach and another requiring the implantation of an intra-aortic balloon counterpulsation device. One patient was on low-dose corticosteroid treatment due to a Giant Cell Arteritis.

### Clinical presentation and neuroimaging

All patients presented with an ischemic vascular event as the primary reason for vascular study that revealed the presence of the thrombus. The clinical presentation included a persistent neurological focality in 8 patients and a transient neurological focality in two patients. However, in all patients the presence of an ischemic lesion was confirmed on MRI in diffusion sequences.

**Figure 1:**
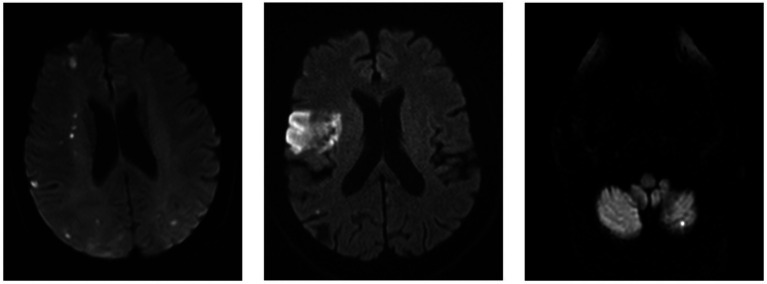
Left: Multiple small scattered pattern in patient 2. Middle: Big isolated infarct in patient 9. Right: Isolated small cerebellar infarct in patient 4.

Regarding the morphology of the ischemic lesion observed on MRI diffusion sequences, more than half of them showed a pattern of small scattered multiterritory infarcts (*n* = 6). The remaining cases showed territorial lesions (*n* = 3) and a punctate lesion in the posterior territory (*n* = 1) (Figure 1).

### Diagnostic method and thrombus characteristics

**Figure 2:**
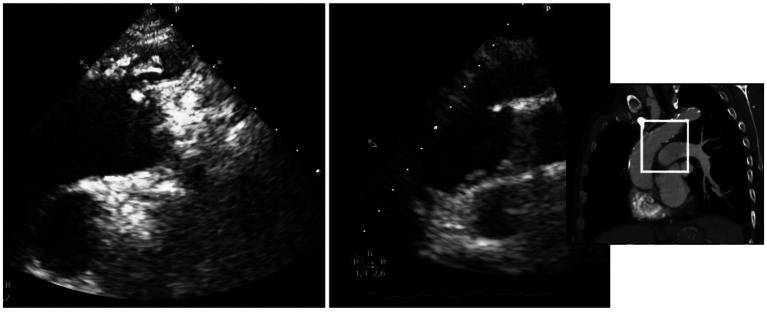
Different morphology of mobile thrombi in the aortic arch, identified using sTTE. Left: unique thrombus attached by a pedunculated portion to an atheroma plaque. Right: Multiple mobile elements suggestive of thrombi and/or plaque fragments compatible con shaggy aorta.

The presence of thrombi was initially identified through sTTE in six patients, with subsequent confirmation by CTA. In the remaining four patients, thrombi were first detected by CTA and subsequently visualized and monitored using sTTE (Figure 2).

**Figure 3:**
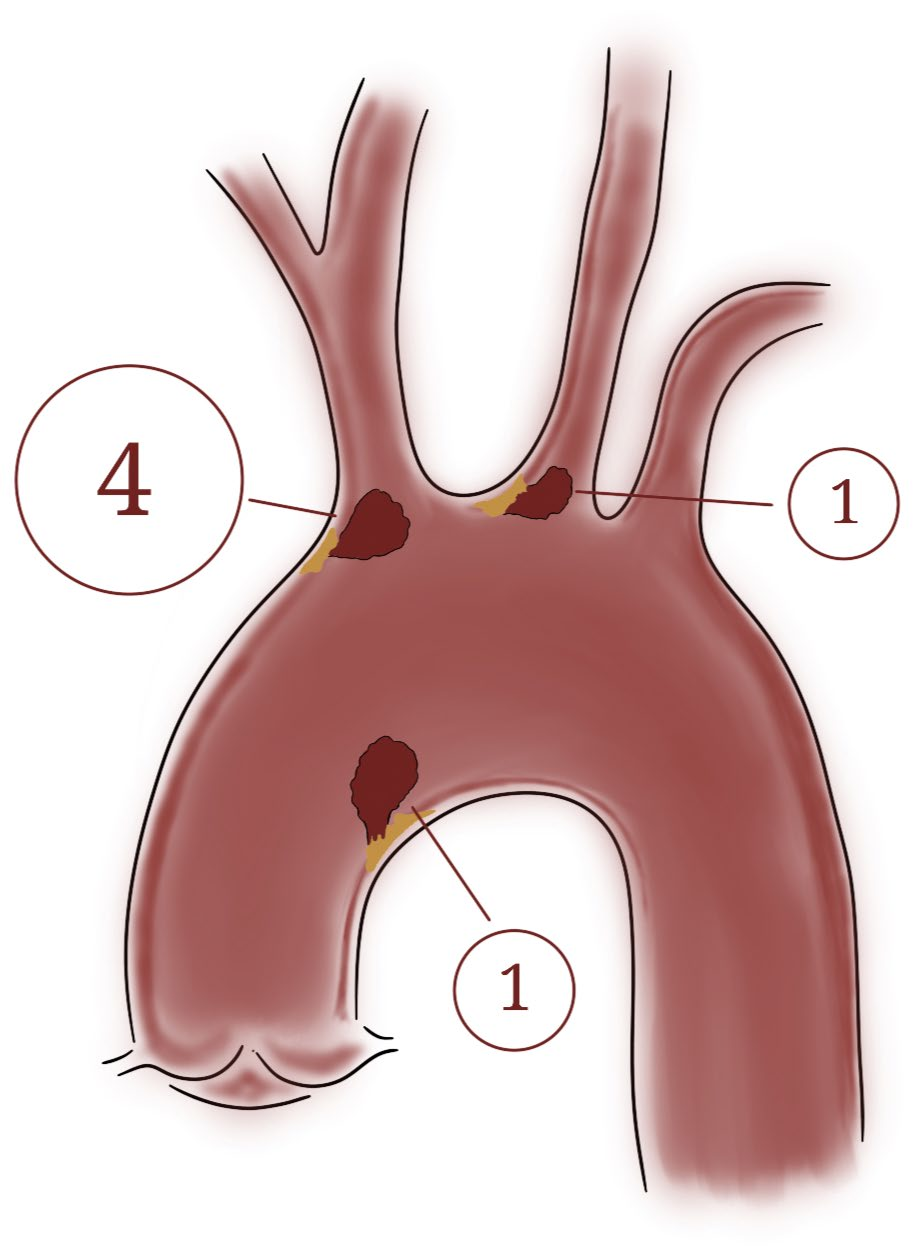
Localization of the six mobile thrombi attached by a pedunculated portion to an atheroma plaque: Origin of the brachiocephalic trunk (*n* = 4), medial wall of the distal portion of the ascending aorta (*n* = 1) and origin of the left common carotid artery (*n* = 1).

In six patients, a pedunculated mobile thrombus was observed. This term describes a blood clot attached to the vascular surface, often at the site of an atheroma or even on an apparently healthy endothelium, via a narrow stalk or pedicle. The designation “mobile” indicates that the thrombus moves or swings freely within the lumen due to blood flow, thereby posing a significant risk of embolization (Supplementary Video S1). The localization was as follows (Figure 3): origin of the brachiocephalic trunk (*n* = 4), medial wall of the distal portion of the ascending aorta (*n* = 1) and origin of the left common carotid artery (*n* = 1).

**Figure 4:**
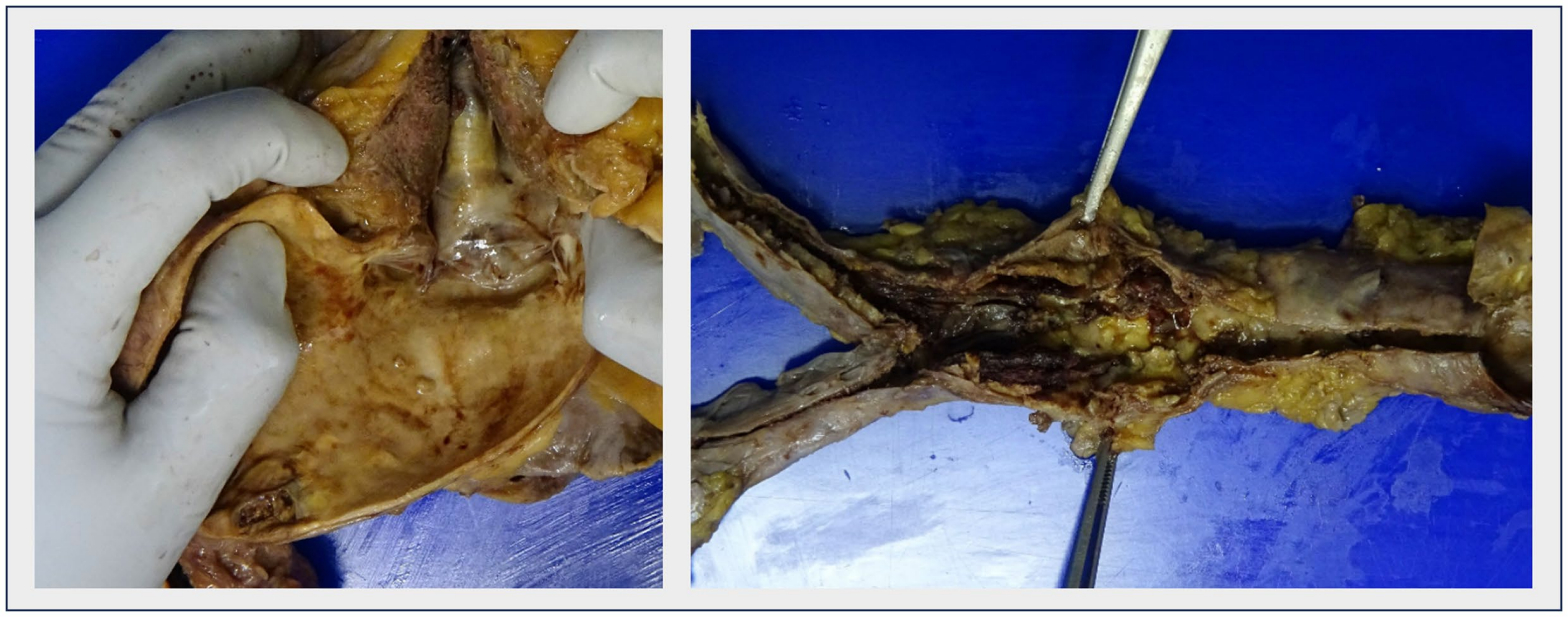
“Shaggy aorta” Severe aortic degeneration, with ulcerated plaques and attached small thrombi. Left: Aortic arch. Right: Abdominal aorta in the same patient.

**Figure 5:**
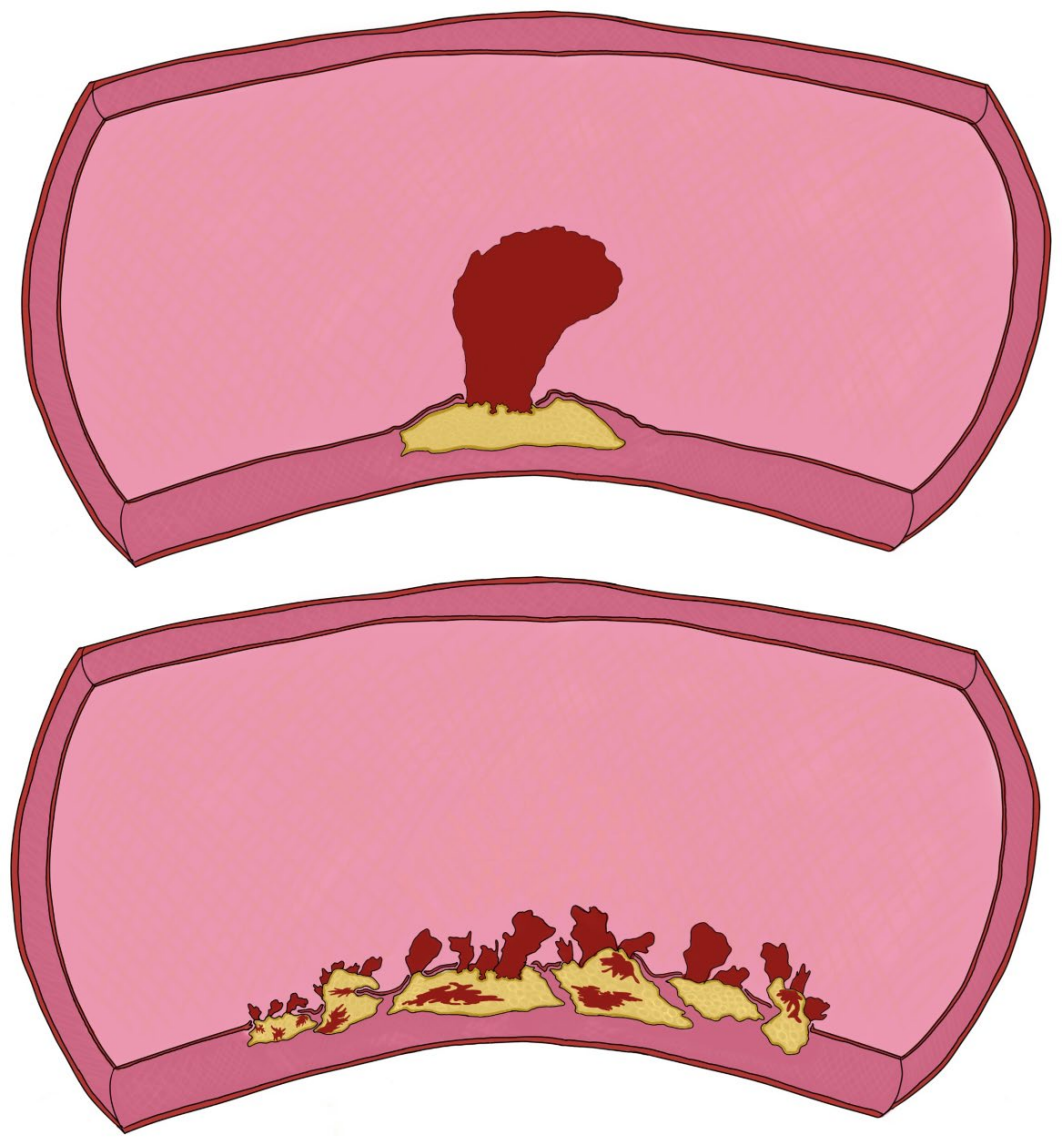
llustration of the two types of mobile thrombi described in this study. Top: Pedunculated mobile thrombus; Bottom: Shaggy aorta.

In four patients, complicated atheromatosis of the aortic arch was identified, with ulcerative lesions and multiple mobile elements suggestive of thrombi and/or plaque fragments, consistent with the “shaggy aorta” pattern (Figure 4; Supplementary Video S2). This term describes the presence of spiculated images visualized through various diagnostic tools, representing an extreme manifestation of aortic atherosclerosis. This condition is characterized by extensive and severe atheromatous disease, featuring scattered ulcers, loosely held debris, a weakened medial arterial layer, and a propensity for thrombus formation (Figure 5) (10).

### Treatment and follow-up

All patients received only medical treatment. Monitoring of all patients was carried out, but three of them died during follow-up. Disappearance of the floating thrombus was observed in 8 patients.

Seven patients were treated solely with an antivitamin K (acenocoumarol). Of them, five exhibited complete thrombus resolution, while two died from renal failure two months post-treatment initiation. One of these patients had a long-standing history of chronic kidney disease stage V, complicated by multiple episodes of acute tubulointerstitial nephritis. The other patient was admitted with acute respiratory failure and developed acute pre-renal failure due to multifactorial causes, including iron-deficiency anemia and a state of dehydration.

One patient maintained treatment with antivitamin K and single antiplatelet therapy (acetylsalicylic acid) concomitantly, showing complete thrombus resolution.

Two patients received triple therapy (anticoagulation plus dual antiplatelet therapy): one with a direct-acting anticoagulant (apixaban) and the other with acenocoumarol, both in combination with acetylsalicylic acid and clopidogrel. Both demonstrated thrombus resolution, but one experienced gastrointestinal hemorrhage, and the other died from hemorrhagic stroke.

During follow-up, stroke recurrences were observed in two of the ten patients. One patient presented with ischemic lesions in both anterior and posterior territories, while the other experienced an anterior hemispheric territorial infarction. Upon confirmation of thrombus resolution with anticoagulant therapy, all patients were transitioned to single antiplatelet therapy and none of them experienced recurrent ischemic stroke.

The clinical presentation, stroke characteristics, treatment and evolution are summarized in Tables 2, 3.

**Table 2:** Diagnosis, thrombus/stroke characteristics and treatment.

*n*	Clinical presentation	Diagnostic method	Thrombus localization	Thrombus morphology	Radiological pattern of the stroke
1	Posterior territory ischemic stroke (POCI)	TTE	Origin of the brachiocephalic trunk	Pedunculated movile thrombus	Small scattered multiterritory
2	Posterior territory ischemic stroke (POCI)	CTA	Origin of the brachiocephalic trunk	Pedunculated mobile thrombus	Small scattered multiterritory
3	Left hemispheric ischemic stroke (PACI)	TTE	Origin of the brachiocephalic trunk	Pedunculated mobile thrombus	Small scattered multiterritory
4	Right hemispheric ischemic stroke (TACI)	CTA	Origin of the brachiocephalic trunk	Pedunculated mobile thrombus	Puntiform
5	Left hemispheric ischemic stroke (PACI)	TTE	Inferior portion of the aortic arch	Pedunculated mobile thrombus	Small scattered multiterritory
6	Left hemispheric ischemic stroke (PACI)	TTE	Origin of the left common carotid artery	Pedunculated mobile thrombus	Small scattered multiterritory
7	Left hemispheric ischemic stroke (TACI)	CTA	Exit of the supraaortic trunks, subclavian arteries, and both carotid arteries	Shaggy aorta	Small scattered multiterritory
8	Right hemispheric ischemic stroke (PACI)	TTE	Aortic arch and descending thoracic aorta, distal to the exit of the supraaortic trunks	Shaggy aorta	Territorial
9	Right hemispheric ischemic stroke (PACI)	CTA	Aortic arch and descending thoracic aorta, distal to the exit of the supraaortic trunks	Shaggy aorta	Territorial
10	Right hemispheric ischemic stroke (PACI)	TTE	Inner wall of the ascending aorta, aortic arch, and descending aorta	Shaggy aorta	Small scattered multiterritory

CTA, Computed Tomography Angiography; TTE, Transthoracic echocardiography.

**Table 3:** Treatment, response, and recurrence.

*n*	Treatment	Treatment response	Stroke recurrence and recurrence pattern	Follow up period (months)
1	Acenocumarol	Resolution	No	7
2	Acenocumarol	Resolution	No	16
3	Acenocumarol	Resolution	No	12
4	Acenocumarol	Resolution	No	84
5	Apixaban Acetylsalicylic acid Clopidogrel	Resolution Digestive hemorrhage	No	7
6	Acenocumarol	Resolution	No	36
7	Acenocumarol	Death due to renal failure	No	2
8	Acenocumarol	Death due to renal failure	Yes, multiterritory	2
9	Acenocumarol Acetylsalicylic acid	Resolution	No	36
10	Acenocumarol Acetylsalicylic acid Clopidogrel	Resolution Death due to hemorrhagic stroke	Yes, territory	8

## Discussion

The present study suggests that sTTE may be a valuable diagnostic and monitoring tool for stroke patients with mobile thrombi in the aortic arch. In addition, oral anticoagulant treatment with acenocoumarol appears to be safe and effective in preventing recurrence of stroke, and the disappearance of the mobile thrombus is achieved.

AAA has been recognized as a significant causal factor in the etiology of stroke (11, 12). Its incidence and severity increase with age, smoking, hypercholesterolemia, hypertension, diabetes mellitus, male sex, hyperhomocysteinemia, or hyperfibrinogenemia. This is a slow and dynamic process with varying degrees of severity, which can progress, regress, or remain stable. The most severe plaques, with the highest embolic risk, are those that are ≥4 mm thick, have mobile components, ulcerated lesions, and hypoechogenic elements (5, 12). In some series, AAA has been detected in up to 29% of patients with embolic strokes of undetermined source (ESUS) and severe AAA in up to 8% (4). However, these numbers might even underestimate the role of AAA as a causal factor, since by considering only patients with ESUS, those with a potentially more severe atherosclerotic profile classified directly as having strokes of atherosclerotic origin are excluded. Additionally, it is possible that a thrombus adhered to the atherosclerotic plaque may have migrated and caused the stroke before being detected.

We recruited 10 patients with mobile aortic arch thrombi associated to AAA. The study is comparable to the series presented by Weiss et al. in 2016, which is, to our knowledge, the only study to date that provides detailed narrative data including risk factors, clinical presentation, and treatment response for this uncommon condition (13). There was a predominance of female patients, consistent with findings from other reported series (14), and exhibited a higher mean age compared to other studies (15). A high prevalence of cardiovascular risk factors was observed, with 70% of patients having at least two recognized factors. Only one patient exhibited a procoagulant abnormality, and in two cases, the stroke occurred following cardiac catheterization. A high embolic risk associated with these types of lesions has been described following procedures such as surgeries or catheterizations, underscoring the importance of characterizing the aorta prior to such interventions (10).

In our series, we identified two distinct types of mobile thrombi: 60% of patients had a pedunculated mural thrombus attached to an isolated atheroma plaque, while the remaining 40% exhibited multiple small thrombi or plaque debris in the context of severe aortic surface degeneration, also referred to as aortic debris or shaggy aorta. The concept of “Shaggy aorta” has been described referring to severe degeneration of the aortic surface, which is extremely friable and can cause systemic embolisms (16). This condition is predominantly observed in elderly patients with severe atherosclerotic disease (10). Aortic atherosclerosis could represent a spectrum of disease, ranging from pure atherosclerotic debris floating in the aorta—most prevalent in elderly patients—to nearly pure clot formations, which are often found in younger patients (17). This hypothesis is supported by pathological examinations of the aortic wall, where the thrombus insertion site consistently involves an atheromatous plaque, even though it may not be visibly calcified (13, 17). However, ‘cryptogenic’ thrombi in the aortic arch have also been described, occurring in the absence of atherosclerotic or aneurysmal disease. These infrequent thrombi have been primarily associated with malignant diseases (infiltration of the aortic wall, paraneoplastic syndrome), prothrombotic conditions such as primary polycythemia vera, antiphospholipid antibody syndrome, hypercoagulable states, primary endothelial disorders, or even iatrogenic causes (18).

Regarding the predominant neuroimaging pattern, as described in recent studies, a small scattered lesion pattern in multiple territories is highly suggestive of AAA (19, 20). This pattern has also been observed in patients with associated cancer (the three territories sign) in various studies, highlighting the importance of investigating this possibility (21). During the follow-up period, none of the patients in our series exhibited this underlying etiology.

The progression of atheromatous plaques and thrombus formation in the aortic arch is a dynamic and evolving process, therefore real-time dynamic imaging techniques can be very valuable for studying these changes. Focused echocardiography allows a rapid approach to the etiological diagnosis of stroke. Its accessibility, bedside applicability, and safety make it an ideal technique for both screening and patient follow-up. Studies have demonstrated that the performance of this technique by appropriately trained neurologists is at least as effective as when conducted in cardiology units (22). Moreover, as previously mentioned, the sTTE plays a significant role in the evaluation of the aortic arch. This view primarily depicts the aortic arch and the three major supra-aortic vessels (innominate, left carotid, and left subclavian arteries), providing a good visualization of the distal ascending aorta, which is a blind spot for TEE (23). So, the most significant advantage over TEE is its higher resolution in the initial portion of the aortic arch, an area where complicated plaques are frequently detected. Furthermore, earlier identification of cardioembolic sources can lead to more timely diagnosis and management of stroke, potentially reducing both the risk of recurrence and the duration of hospitalization, consequently mitigating the economic burden of stroke (22).

The optimal management strategy for aortic arch thrombi remains unclear. A variety of approaches are currently employed, including conservative treatments with anticoagulation or thrombolysis, interventional modalities such as thromboaspiration or balloon-catheter thrombectomy, and open surgical procedures such as thrombectomy, thromboendarterectomy, and aortic prosthetic replacement (24). While oral anticoagulation is generally considered the initial treatment, early surgical intervention has shown promising results. Both endovascular and open surgical techniques have demonstrated safety, suggesting that definitive treatment should be tailored to individual patient characteristics (25). Some authors have even suggested that symptomatic individuals should be classified as high-risk for recurrent embolism and consequently, they should undergo surgical removal, reserving conservative treatment for selected cases such as high-risk and elderly patients with contraindications for surgery, as well as asymptomatic individuals (13).

The recently published 2023 European Society for Vascular Surgery (ESVS) Guidelines (26) have attempted to provide guidance on the management of mobile carotid thrombi, strongly recommending anticoagulation (Class I, Level C). Given the lack of evidence on the optimal management of floating thrombi in the aortic arch, and based on the analogy with carotid floating thrombi, we decided to initiate empirical treatment with anticoagulation once the presence of a floating thrombus was confirmed.

It was observed a positive response to anticoagulation therapy in the majority of patients, with most achieving complete thrombus resolution. Notably, vitamin K antagonists, both as monotherapy and in combination with antiplatelet agents when previously prescribed, proved effective in resolving thrombi. However, the efficacy of these treatments must be weighed against potential risks. Hemorrhagic complications were observed in two patients. Of particular concern was the occurrence of a fatal hemorrhagic stroke in one patient receiving triple therapy, while another patient on the same regimen experienced non-fatal gastrointestinal bleeding. These observations underscore the delicate balance between achieving therapeutic anticoagulation and minimizing bleeding risks, especially in complex treatment regimens.

Upon confirmation of mobile thrombus resolution, anticoagulation was discontinued in all cases, with no subsequent ischemic recurrences observed. Notably, the three patients who died during follow-up (two from renal failure and one from cerebral hemorrhage) and the two who experienced stroke recurrence prior to thrombus resolution presented with aortic debris, a phenotype associated with advanced age and an increased burden of cardiovascular risk factors (16). Interestingly, in our series, patients with this type of aortic involvement had a lower mean age than those with a single pedunculated thrombus (67.2 vs. 81.5 years), although they exhibited a higher prevalence of cardiovascular risk factors (particularly a greater incidence of dyslipidemia and smoking).

It is important to note that this study is limited by its observational design and the absence of a control group. Patients were included consecutively as they were identified with mobile thrombi following an initial ischemic event. Although some studies have implemented methodological procedures to mitigate selection bias, most aortic plaque studies only include patients who are symptomatic and subsequently referred for diagnostic evaluations. Consequently, these studies, including the present one, are not entirely free from such biases. Furthermore, a direct comparison between transthoracic echocardiography through the suprasternal window and the angioCT was not conducted. As a result, no conclusions can be drawn in this regard, as this is beyond the scope of the study. Regarding treatment, there are no universal recommendations in the literature for selecting the most appropriate therapeutic modality for each patient. The limited sample size of this study precludes drawing generalizable conclusions about treatment modalities. Additionally, there are currently no established guidelines for the follow-up of these patients, and significant variability exists in the study. Patients were included prospectively as they met the selection criteria, resulting in some patients being studied for several months while others had shorter follow-up periods.

## Conclusion

In conclusion, our findings suggest that sTTE may serve as a valuable technique for the detection and monitoring of mobile thrombi in the aortic arch, as it is an accessible and less invasive method compared to current reference tests. Therefore, it is essential to conduct diagnostic and therapeutic utility studies specifically designed for this purpose, in order to confirm this hypothesis and recommend its implementation. Nevertheless, given its accessibility and safety for patients, we propose the implementation of this technique in clinical practice as a screening method to identify potential embolic sources in patients presenting with embolic stroke, particularly when a pattern of multiple scattered cortical lesions in different areas is observed. Finally, until additional studies provide adequate evidence, early anticoagulation may be an effective and safe treatment option for some patients. However, further research is warranted to optimize treatment protocols and identify patient factors that may predispose to complications, thereby improving overall outcomes in this high-risk population.

## Data Availability

The raw data supporting the conclusions of this article will be made available by the authors, without undue reservation.

## Supplementary material

SUPPLEMENTARY VIDEO S1 Suprasternal window, axial view at the level of brachiocephalic trunk, showing a mobile thrombus attached to an hyperechogenic atherosclerotic plaque.

SUPPLEMENTARY VIDEO S2 Suprasternal window, longitudinal view, showing an aortic arch with irregular surface at inferior wall, produced by multiple small atherosclerotic plaques with several mobile components attached.
